# Compiling a versatile toolbox for inducible gene expression in *Methanosarcina mazei*

**DOI:** 10.1093/femsml/uqae019

**Published:** 2024-10-10

**Authors:** Johanna Hüttermann, Ruth Schmitz

**Affiliations:** Institute for General Microbiology, Christian-Albrechts-University, Am Botanischen Garten 1-9, 24118 Kiel, Germany; Institute for General Microbiology, Christian-Albrechts-University, Am Botanischen Garten 1-9, 24118 Kiel, Germany

**Keywords:** inducible gene expression, antisense RNA-dependent knockdown, RNA thermometer, selection marker, streptothricin acetyltransferase, nourseothricin

## Abstract

*Methanosarcina mazei* is a model organism, providing a platform to explore methanoarchaeal regulation mechanisms on the transcriptional and translational level. This study investigates and evaluates various molecular tools to allow inducible gene expression in *M. mazei*. (i) The TetR/TetO system was utilized to induce expression of a designed antisense RNA directed against sRNA_154_ allowing to increase transcripts of asRNA_154_ (500-fold), resulting in a significant decrease of sRNA_154_ levels (tetracycline-induced knockdown mutant). Strong reduction of sRNA_154_ was further confirmed in the knockdown mutant by up to 50-fold decreased transcript levels of the genes *nifH, glnK_1_*, and *glnA_1_*, the stability of which is increased by sRNA_154_. (ii) For translational regulation, an RNA thermometer was designed and first-ever utilized in an archaeon, inserted into the 5′-untranslated region of a reporter gene, which showed enhanced protein expression upon a temperature shift from 30°C to 40°C. (iii) The long 5′-UTR of a trimethylamine (TMA)-inducible polycistronic mRNA was evaluated and studied as a potential genetic tool for induced gene expression on the translational level. However, we discovered TMA-dependent regulation occurs most likely on the transcript level. (iv) A new selection marker (nourseothricin resistance) was established for *M. mazei* using the streptothricin acetyltransferase gene. Taken together, our findings provide a foundation for future exploration of genetic regulation and inducible gene expression in *M. mazei* and other methanoarchaea, advancing genetic studies in these organisms and enhancing their potential for biotechnology applications.

## Teaser

Various molecular tools are presented for controlling gene expression in *Methanosarcina mazei* at both the transcriptional and translational levels. Gene regulation is achieved, for example, through the use of an engineered tetracycline inducible promoter to modulate transcription and an RNA thermometer to control translation. These tools enable precise manipulation of gene expression, offering insights into regulatory mechanisms in methanogenic archaea. Key steps in both transcriptional and translational control are highlighted, demonstrating the versatility and effectiveness of these approaches.

## Introduction

*Methanosarcina mazei* is a key model organism for studying methanogenic archaea due to its robust genetic tractability and its distinctive metabolic pathways. Some established genetic tools accessible for *Methanosarcina* species have enabled in-depth investigation into the physiology, genetics, and metabolic processes of this ecologically important methane-producing archaeon (Enzmann et al. 2018). For instance, a completely sequenced genome (Deppenmeier et al. 2002), a DNA transformation system (Ehlers et al. 2005), precise markerless chromosomal gene deletions (Ehlers et al. 2005, Thomsen et al. 2022), and an optimized shuttle vector for protein expression (Thomsen and Schmitz 2022) have been recently reported for *M. mazei*.

### Inducible gene expression in methanoarchaea

Despite all the progress in archaeal genetics, the exploitation of inducible gene expression systems lacks behind in archaea. While the strength and adaptability of inducible gene expression are widely appreciated and has extensive availability in bacterial and eukaryotic hosts, similar systems for archaea are still rare and are being optimized (Guss et al. 2008, Mondorf et al. 2012, Farkas et al. 2013, Demolli et al. 2014). Until now, common overexpression systems mostly use a strong promoter placed upstream of the target gene, even though inducible systems with a dose-dependent response are more appropriate for studies requiring induced expression, e.g. for toxic proteins (Farkas et al. 2013, Contreras et al. 2022). The first artificially designed promoter in archaea independent of growth conditions was the tetracycline-inducible expression system using an operator site for the bacterial repressor TetR (Guss et al. 2008). Although protein-mediated control of gene expression is well appreciated since many decades, post-transcriptional RNA-based regulation has been discovered in the early 2000s as a complementary level of regulation. Small regulatory RNAs have emerged as one of the most widespread and important gene regulatory systems in bacteria (Wagner and Romby 2015), highlighting their importance in gene expression control (Sharma et al. 2022). Thus, post-transcriptional regulation offers an alternative approach, involving either *cis*-acting elements or *trans*-regulatory RNAs (Roßmanith and Narberhaus 2016, Contreras et al. 2022). The application of *cis*-acting RNAs has become an effective method for inducing translation in bacteria, haloarchaea, and Thermococci (Speed et al. 2018, Born et al. 2021). In contrast, methanogenic archaea have been limited to a few *cis*-acting systems, such as the artificially designed tetracycline-responsive riboswitch (Demolli et al. 2014), and RNA thermometers (RNATs) have not yet been employed in archaea. RNATs, always located in the 5′-UTR of an mRNA and 5′UTR riboswitches, both regulate translation by forming secondary structures that often block the ribosome-binding site (RBS) or start codon. Riboswitches respond to specific ligands, inducing structural changes, while RNATs respond to temperature changes that alter RNA conformation (Roßmanith and Narberhaus 2016). On the other hand, *trans*-regulatory RNAs (Xie et al. 2021) were already discussed to possess significant potential to enhance a wide range of inducible gene expression systems in methanogenic archaea (Contreras et al. 2022), even though not applied until now. However, several noncoding regulatory RNAs have been investigated in *M. mazei* such as sRNA_154_ (Jäger et al. 2009). sRNA_154_ is the central regulatory sRNA in nitrogen (N) metabolism in *M. mazei* with functions ranging from positive post-transcriptional regulation to inhibition of translation initiation (Prasse et al. 2017).

### A new selection marker for *M. mazei*

The utilization of the abovementioned genetic techniques requires the use of a selectable marker, typically an antibiotic resistance gene, to distinguish cells that have acquired desired genetic alterations from those that have not undergone transformation. Genetic methodologies in methanogenic archaea primarily rely on puromycin resistance mediated by the *pac* gene from *Streptomyces alboniger* (Gernhardt et al. 1990, Farkas et al. 2013). Puromycin is an antibiotic that mimics the 3′-end of aminoacyl-tRNA, binds to the A site in the ribosome, and causes premature chain termination during protein synthesis. The *pac* gene encodes puromycin *N*-acetyltransferase, an enzyme that inactivates puromycin by acetylating and blocking the reactive amino group in puromycin (Aviner 2020). Selections based on resistance to neomycin (Argyle et al. 1996, Mondorf et al. 2012) and pseudomonic acid (Boccazzi et al. 2000) are also utilized but to a limited extent. Neomycin, an aminoglycoside antibiotic, interferes with prokaryotic ribosomes during the translation process, specifically targeting the decoding site on the 16S rRNA of the 30S ribosomal subunit (Foster and Champney 2008). The *aph-IIb* gene encodes aminoglycoside phosphotransferase, an enzyme that phosphorylates aminoglycoside antibiotics like neomycin, which makes it unable to bind to its target (Kim and Mobashery 2005). Pseudomonic acid inhibits isoleucyl-tRNA synthetase, thereby disrupting protein synthesis, and mutations in the *ileS* gene can lead to a form of isoleucyl-tRNA synthetase that is resistant to inhibition by pseudomonic acid (Boccazzi et al. 2000). However, pseudomonic acid is not frequently used due to higher costs, limited commercial availability, and the large size of the *ileS* gene (Farkas et al. 2013). After its broad applicability across bacteria and eukarya, nourseothricin in combination with the streptothricin acetyltransferase (*sat*) gene from *Streptomyces rochei* was recently reported to be effective in *Methanosarcina acetivorans* and *Methanosarcina barkeri* (Farley and Metcalf 2019). Nourseothricin, comprising streptothricins C, D, E, and F, is a commercially available antibiotic that disrupts protein translation by inducing mRNA miscoding in bacteria and eukarya (Haupt et al. 1978, 1980). The streptothricin acetyltransferase has been demonstrated to confer nourseothricin resistance by inactivating nourseothricin through monoacetylation of its β-lysine residue. Hence, the *sat* gene with nourseothricin can serve as a selection marker for genetic modification of recombinant strains, which has been reported in various organisms, including Gram-positive and Gram-negative bacteria, yeast, filamentous fungi, protozoa, microalgae, plants, and archaea (Horinouchi et al. 1987, Joshi et al. 1995, Jelenska et al. 2000, Reuss et al. 2004, Alshahni et al. 2010, Ifuku et al. 2015, Farley and Metcalf 2019, Yang et al. 2019).

Here, we report of the investigation of different inducible expression systems. (i) The TetR/TetO system (Guss et al. 2008) that was now adapted for *M. mazei* and was used to overexpress an antisense RNA to generate a tetracycline inducible knockdown mutant. (ii) We engineered an RNAT to enable temperature inducible protein production in archaea, to our knowledge marking the first instance of its kind. (iii) We gained new insights into the reported trimethylamine (TMA)-inducible expression (Mondorf et al. 2012). (iv) We report of a new selection marker in *M. mazei* with the nourseothricin-*sat* gene pair adapted from *M. acetivorans* (Farley and Metcalf 2019).

## Materials and methods

### Strains and plasmids

All the strains and plasmids used in this study are listed in Table 1. The transformation of plasmid DNA into *M. mazei* 3A was conducted through liposome-mediated transformation as described by Ehlers et al. (2005) and modified by Gehlert et al. (2023). Additionally, plasmid DNA was introduced into *Escherichia coli* DH5α, JM109 λpir, or BL21-CodonPlus^®^-RIL, using the method described by Inoue et al. (1990).

**Table 1.** Strains and plasmids used in this study.

Strain/plasmid	Genotype/relevant characteristics	Source/reference
*M. mazei* strain Gö1	Wild type	DSM number 3647
*M. mazei* 3A	Potential cell wall mutant	Ehlers et al. (2005)
*E. coli* DH5α	General cloning strain	Miller and Mekalanos (1988)
*E. coli* JM109 λpir	General cloning strain	Miller and Mekalanos (1988)
BL21-CodonPlus®®-RIL	General expression strain	Stratagene, La Jolla, USA
pET21a(+)	General expression vector	Novagen, Darmstadt, Germany
pCRII-TOPO	General cloning vector	Invitrogen, Darmstadt, Germany
pWM321	Shuttle vector *M. mazei*/*E. coli* oriR6K:pC2A replicon	Metcalf et al. (1997)
pGK050	Template for *tetR* amplification	Guss et al. (2008)
pRS651	pCR2.1+ synthesized p_tetO1_	This study
pRS893	pDrive/p*_mcrB_*	Ulbricht et al. (2020)
pRS1271	pWM321 + p_tetO1_	This study
pRS1534	Cloning vector pUC57 + synthesized *sat* gene and codon optimized for *M. acetivorans*	provided from Dr Michael Rother, Dresden, Germany
pRS1542	pWM321 + *sat* gene	This study
pRS1544	pWM321 + p_tetO1_ + *tetR*	This study
pRS1555	pWM321 + p_tetO1_ + *tetR* + asRNA_154_	This study
pRS1595	Optimized shuttle vector *M. mazei*/*E. coli* oriR6K:pC2A replicon	Thomsen and Schmitz (2022)
pRS1665	Optimized shuttle vector ∆*Nde*I restiction site	This study
pRS1797	Cloning vector + synthesized 5′-UTR of *MM_1687*	This study
pRS1807	pRS1665 + p*_mcrB_*	This study
pRS1809	Expression vector pET21a(+) + *glnK_1__his_6_*	This study
pRS1826	pRS1595 + p*_mcrB_* + *glnK_1__his_6_*	This study
pRS1893	Cloning vector + synthesized 5′-UTR of *MM_1687*	This study
pRS1913	pRS1665 + 5′-UTR of *MM_1687* + *glnK_1__his_6_*	This study
pRS1931	TOPO + synthesized p*_mcrB_* + RNAT No. 1 + *glnK_1__his_6_*	This study
pRS1935	TOPO + synthesized p*_mcrB_* + RNAT No. 5 + *glnK_1__his_6_*	This study
pRS1939	TOPO + synthesized p*_mcrB_* + RNAT No. 9 + *glnK_1__his_6_*	This study
pRS1958	pRS1595 + synthesized p*_mcrB_* + RNAT No. 1 + *glnK_1__his_6_*	This study
pRS1962	pRS1595 + synthesized p*_mcrB_* + RNAT No. 5 + *glnK_1__his_6_*	This study
pRS1966	pRS1595 + synthesized p*_mcrB_* + RNAT No. 9 + *glnK_1__his_6_*	This study

### Generation of plasmids

The construction of the plasmid pRS1555 (see Fig. S6) containing the antisense RNA_154_ under the tetracycline-inducible promoter p_tetO1_ and the *tetR* gene under the constitutive promoter p*_mcrB_* proceeded as follows: first, the promoter p_tetO1_ was amplified from pRS651 using specific primers (5′-TTTACTAGTGCATGCTTCATTTATC-3′ and 5′-AAAGAGCTCAATCTCTATCACTG-3′) and introduced in pWM321 via SpeI/SacI restriction sites. Next, the *tetR* gene under the promoter p*_mcrB_* was amplified from pGK050 with specific EcoRV restriction sites (5′-TTTGATATCCTGGGGGTACCGAAGTTC-3′ and 5′-GTTGATATCCACAGGAAACAGCTATGACC-3′) and subsequently cloned into pRS1271. Lastly, the asRNA_154_ was amplified from genomic DNA of *M. mazei* DSM number 3647 using specific primers (5′-GTTGCTCACGAGCTCAACGTCAG-3′ and 5′-AGAATTTCTCGGTACCAAAAG-3′), followed by SacI/KpnI restriction and T4 ligation in pRS1544.

To allow cloning with NdeI restriction enzymes, the NdeI restriction site in *ORF1* of the optimized shuttle vector pRS1595 was mutated via silent point mutation introduced via site-directed mutagenesis using specific primers (5′-CACATGTGTATAAGGTGCTATATC-3′ and 5′-CACATGTGTGATTTTTTAGTAGTC-3′) leading to pRS1665. As a positive control the constitutive promoter p*_mcrB_* was amplified from pRS893 with primers introducing XhoI/NdeI restriction sites (5′-CAGATGAGCTCGAGCCCTAAAAATTAAATTTTC-3′ and 5′-CCATCATATGATTTCCTCCTTAATTTATTAAAATC-3′) and subsequently cloned into pRS1665. Since GlnK_1_ served as a read out, its gene was amplified from gDNA of *M. mazei* DSMZ number 3647 utilizing customized primers (5′-TGGTGGTCCATATGAAATACGTAATTGCAATG-3′ and 5′-CAACCTCGAGAATTGCCTCAGGTCCGG-3′) and cloned in the expression vector pET21a(+). Next, the gene was amplified from pRS1809 together with a his-tag and a terminator sequence employing specific primers (5′-GAAGGAGATATACATATGAAATACGTAATTGC-3′ and 5′-TGTTGCTAGCAAAAAATCAGTGGTGGTGGTGGTGG-3′) and introduced under the control of the p*_mcrB_* in pRS1807 using NdeI/NheI restriction sites resulting in pRS1826.

The RNATs were synthesized as gene fragments (Twist Bioscience, South San Francisco, CA, USA) and were polymerase chain reaction (PCR) amplified using specific primer sets (5′-GTCAGGCCGGCCCTCGAG-3′ and 5′-CAGTGGCCGGCCGCTAGC-3′). The resulting PCR products were TA-cloned into pCRII-TOPO resulting in plasmids pRS1931-39. Next, these different 5′-untranslated regions (5′-UTR) with a *glnK_1__his_6_* reporter gene were introduced in pRS1595 using XhoI/NheI restriction sites. To test TMA induction, the 5′-UTR of *MM_1687* was synthesized (Twist Bioscience), flanked by a XhoI and a NdeI restriction site and introduced in pRS1665. Next, *glnK_1__his_6_* reporter gene was cut from pRS1826 and incorporated via NdeI/NotI restriction sites and T4 ligation leading to pRS1913. The 26 nt sequence (see Table S1) originates from the 5′-UTR of the heat shock gene *dnaJ* from *Brucella melitensis* (Waldminghaus et al. 2007).

To test the *sat* gene as a positive selection marker under nourseothricin pressure the *sat* gene under the control of the p*_mcrB_* promoter was synthesized (BioCat GmbH, Heidelberg, Germany; kindly provided from AG Michael Rother, TU Dresden) and incorporated in pWM321 using SbfI restriction enzyme. All constructs were verified by sequence analysis.

### Growth of *M. mazei*

*Methanosarcina mazei* strains were cultivated anaerobically without shaking at 37°C if not stated otherwise. All temperature dependent experiments were performed in water baths. Cultures were incubated with an N_2_ and CO_2_ (v/v, 80/20) atmosphere in either 5 ml or 50 ml minimal medium within sealed Hungate tubes or serum bottles, respectively, containing 75 mM methanol as the sole energy and carbon source if not stated otherwise (Ehlers et al. 2005). To prevent bacterial contamination, 100 µg/ml ampicillin was added, while 5 µg/ml puromycin was added for plasmid selection. Growth progress was typically monitored by measuring the optical density of the cultures at 600 nm. To determine the minimal inhibitory concentration (MIC) of tetracycline and nourseothricin, the final optical density at 600 nm (OD_600_) of the cultures was measured after 4–7 days. For testing temperature induction, precultures were already grown at the respective temperatures. After the inoculation of the main cultures, they were cultivated until reaching the early exponential phase (OD_600_ of 0.3–0.4). Cultures were then split in two cultures and were incubated for 1 h. After this recovery time half of the cultures were transferred to a higher temperature and incubated for 2.5 h. To test TMA induction, the cultures were also grown to early exponential phase with 30 mM methanol as carbon source, before induction by addition of 50 mM TMA. The cultures were then harvested as described before (Veit et al. 2005). Parts of the cell pellet was used for RNA preparation, while the rest was prepared for western blot analysis.

### Cell extract preparation and western blot analysis

For western blot analysis, crude cell extracts were prepared. Exponentially growing cultures were harvested and cells were resuspended in phosphate buffered saline (137 mM NaCl, 1.5 mM KH_2_PO_4_, 7.8 mM Na_2_HPO_4_, 2.7 mM KCl, and pH 7.4) and disrupted using a Geno/Grinder® (1300 strokes, 6 min). The whole cell extract was centrifuged for 30 min at 13 000 × *g* and 4°C to remove cell debris and the remaining unlysed cells. Total protein concentration of the supernatant was determined using Bradford reagent and samples were supplemented with 2x SDS loading buffer and boiled for 15 min. 5 or 10 µg of whole cell extract was loaded on the SDS-PAGE for experiments regarding temperature induction and TMA induction, respectively, followed by western blotting. The blot was probed with anti-His tag primary antibody (Qiagen, Venlo, Netherlands), followed by goat antimouse IgG–HRP conjugate secondary antibody (Bio-Rad Laboratories, Inc., Hercules, CA, USA). The signal was detected using chemiluminescence (SuperSignal^™^ West Femto Maximum Sensitivity Substrate from Thermo Fisher Scientific, Darmstadt, Germany) and visualized using a Chemidoc (Bio-Rad Laboratories, Inc.). Protein band intensity was quantified using Image Lab (Bio-Rad Laboratories, Inc.), and data were calculated from a standard curve. For a standard curve, GlnK_1_ was purified from an *E. coli* BL21-CodonPlus^®^-RIL overexpression strain using a his-tag and Ni-NTA purification as described before (Habenicht et al. 2023).

### RNA preparation and qRT-PCR analysis

For quantitative reverse transcription (qRT)-PCR analysis, 5 or 25 ml *M. mazei* cultures were rapidly cooled down, harvested at 4000 × *g* at 4°C for 30 min and cell pellets resuspended in ROTI^®^Zol (Carl Roth, Karlsruhe, Germany). Total RNA was isolated by chloroform extraction and was followed by DNase I treatment and phenol–chloroform precipitation (Veit et al. 2005). qRT-PCR was performed using the QuantiTect SYBR Green RT-PCR Kit (Qiagen, catalogue number 204243) according to manufacturer’s instructions and as described before (Veit et al. 2005). 40 ng/µl total RNA from *M. mazei* cultures, the in Table 2 listed primers and the ViiA 7 real-time PCR system from Applied Biosystems (Thermo Fisher Scientific) were used for qRT-PCR. Ct values were normalized in respect to the corresponding Ct values obtained from the same RNA for three housekeeping genes (MM_1215, MM_1621, and MM_2181). The fold change was calculated using the formula fold change equals 2^−∆∆Ct^, as described before (Veit et al. 2005).

**Table 2.** Primer sets used for quantitative RT-PCR analysis.

Target	Forward primer	Reverse primer
qRT-PCR MM_1215	5′-TCAAGAGCGAGGGCATGAATG-3′	5′-GCACTACCGAGAACAATAGCC-3′
qRT-PCR MM_1621	5′-TAGGAGGTTTTCTCGGAAGCG-3′	5′-AAGCGTATCTCCATCAAGCCC-3′
qRT-PCR MM_2181	5′-GCCTCCATGAGAAGAATGCTC-3′	5′-CTTCAAGGTCTCCAACTCCTG-3′
qRT-PCR MM_sRNA154	5′-CCGGTTGCTTACGAGTAAATC-3′	5′-TGAAGGAAGTTAGTTGCTCACG-3′
qRT-PCR MM_asRNA154	5′-GAGCTCAACGTCAGGAACG-3′	5′-TCGGTACCAAAAGTTTTAAATAGAAATATACG-3′
qRT-PCR MM_0719	5′-CCACGCAGAATCTTACTG-3′	5′-AGCACGGTTTTCTGGTTC-3′
qRT-PCR MM_0732	5′-CGATGGAATATGATGCAAACC-3′	5′-CCAACGTAACCGTCACTG-3′
qRT-PCR MM_0964	5′-GGGAGGATACTTCGATTTCG-3′	5′-TGATGGGAGGCTTCTATCTG-3′
qRT-PCR glnK_1__his_6_	5′-GATCGAAATCGCTGTAAATGAC-3′	5′-GGTGGTGCTCGAGAATTG-3′
qRT-PCR MM_1687	5′-CGGTCGTGATGTCCCGATCAGGAAC-3′	5′-CCGCCGATCATGACCTTTACTTTGTCTC-3′

## Results and discussion

To enhance protein expression in *M. mazei* several innovative approaches have been pursued in this study, including the development of novel inducible expression systems on the transcriptional and translational level. Additionally, we introduced a new selection marker in *M. mazei* using the streptothricin acetyltransferase (*sat*) gene from *S. rochei* (Farley and Metcalf 2019). These advancements not only broaden the genetic toolbox for fundamental research in the model *M. mazei*, such as investigating essential genes with inducible antisense systems or complementing knockout genes using the *sat* gene, but also strengthen basic research in methanogens in general.

### Established tetracycline-inducible antisense RNA for sRNA knockdown

**Figure 1.**
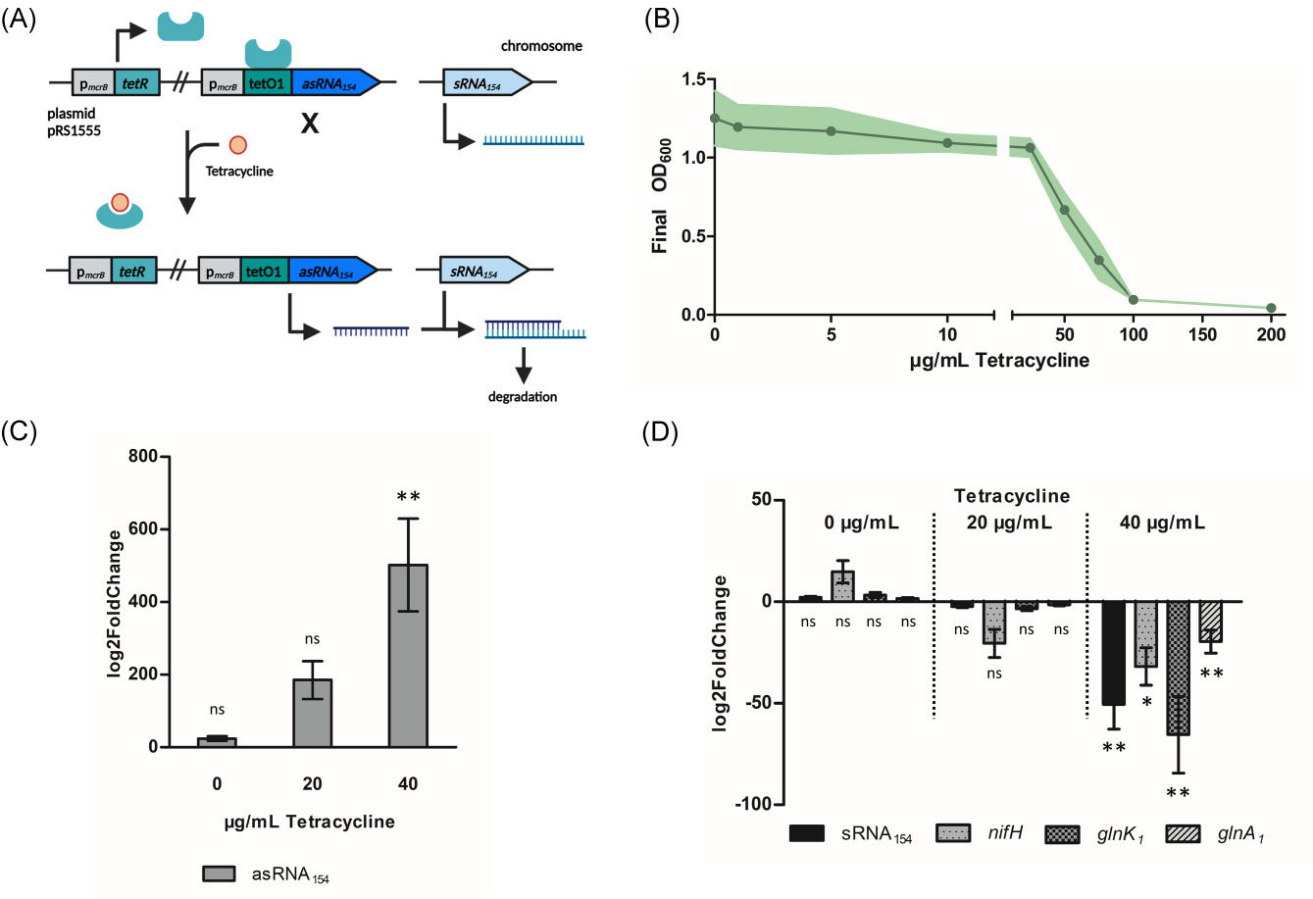
Tetracycline-inducible gene expression in *M. mazei* using TetR and the tetO1 element. (A) Overview of the regulation strategy: tetracycline induces antisense RNA_154_, which is complementary to sRNA_154_, resulting in sRNA_154_ degradation creating an inducible knockdown mutant. (B) To determine the inhibitory effect of tetracycline, 5 ml *M. mazei* wild type (DSMZ 3647) was grown with varying tetracycline concentrations (0–200 µg/ml) with final optical density determined after 4–5 days. Data represent mean values from three biological replicates; standard deviations shown as shaded areas. (C) and (D) show fold change of mRNA expression under N deficiency before and after induction with 20 and 40 µg/ml tetracycline, compared to a not induced empty vector control. Fold change in expression of (C) the induced asRNA_154_, and (D) the in consequence downregulated transcripts sRNA_154_, *nifH, glnK_1_*, and *glnA_1_* in the *M. mazei* mutant carrying pRS1555. Values were normalized with three housekeeping genes, and fold change calculated using 2^−∆∆Ct^ as described in the section ‘Materials and methods’. Data represent mean values from two biological replicates with at least two technical replicates each, standard deviations are shown as error bars. Significance was calculated using a one-way ANOVA followed by a *post hoc* Tukey test with *P*-values resembled as stars with P** < .01, P* < .05, and ns = not significant using GraphPad Prism version 9.4.1 for Windows, GraphPad Software, San Diego, CA, USA, www.graphpad.com (accessed on 3 May 2024). Partially created with BioRender.com.

First, the TetR/TetO system was used for tetracycline-regulated gene expression on the transcriptional level. Therefore, the constitutive promoter p*_mcrB_* was modified to include a binding site (tetO1) for the TetR protein as described before (see Fig. 1A; Guss et al. 2008). In the absence of tetracycline, TetR represses target gene expression. Upon tetracycline addition, TetR binds tetracycline, altering its conformation and allowing gene expression (see Fig. 1A). Initially, the ability of *M. mazei* to survive increasing tetracycline concentrations was tested (see Fig. 1B). The results demonstrate that the organism can tolerate up to 25 µg/ml tetracycline without strong effects regarding its growth. Unfavourably, 100 µg/ml tetracycline, which was used to induce gene expression in *M. acetivorans* (Guss et al. 2008), almost completely inhibits the growth of *M. mazei*. Consequently, 20 and 40 µg/ml were used to test the inducible expression system in *M. mazei*.

The well-described *trans*-encoded sRNA_154_ (Prasse et al. 2017) was used for proof of concept. sRNA_154_ is exclusively expressed under N deficiency and has been shown to have a crucial regulatory role in the N metabolism of *M. mazei* by stabilizing e.g. the polycistronic mRNA encoding structural genes of the nitrogenase and the glutamine synthetase (Jäger et al. 2009, Prasse et al. 2017). Here, the expression of antisense RNA_154_, which is complementary to sRNA_154_, led to the formation of double-stranded RNA that was subsequently degraded by RNases, creating a tetracycline-inducible sRNA_154_ knockdown mutant (see Fig. 1A). Due to the effect of tetracycline to *M. mazei* growth (Fig. 1B), the growth phenotype of this knockdown mutant cannot be directly compared to the existing sRNA_154_ deletion strains (Ehlers et al. 2011, Prasse et al. 2017). However, as sRNA_154_ has positive stabilizing function in the N metabolism of the cells under N deficiencies, several of the transcripts associated with the N fixation and metabolism like *nifH, glnK_1_*, and *glnA_1_* are stabilized by sRNA_154_ and thus should also be downregulated upon asRNA_154_ induction. To test this prediction the transcript levels of asRNA_154_, sRNA_154_, and the target genes of sRNA_154_ were assessed using qRT-PCR. Our results demonstrate that the amount of asRNA_154_ transcript increases almost linear to the tetracycline concentration with a maximal change of about 500-fold compared to a not induced empty vector control (see Fig. 1C). The findings presented herein are congruent with the results reported by Guss et al. (2008), wherein induction was maximal in *M. acetivorans* with tetracycline concentrations exceeding 33 µg/ml. Furthermore, the magnitude of induction observed in this study is comparable to previous findings, despite the disparity in measurement methodologies; whereas protein activity was quantified in previous studies (Guss et al. 2008), the present investigation evaluates transcript levels. Interestingly, the transcript levels of sRNA_154_, *nifH, glnK_1_*, and *glnA_1_* were only slightly affected at 20 µg/ml tetracycline but showed a significant downregulation in the presence of 40 µg/ml tetracycline of about 50-fold (see Fig. 1D). Notably, the transcript for the artificial asRNA_154_ was already upregulated ~24-fold prior induction compared to a not induced empty vector control. This leakiness of the promoter can be minimized by using multiple repressor-binding sites like described before, but would come at a cost of lower induction rates (Guss et al. 2008). This basal expression of asRNA_154_ may prompt cellular adaptation under N deficiencies, resulting in increased transcript levels of sRNA_154_ around 2-fold, along with upregulation of associated transcripts such as *nifH* by ~15-fold. This upregulation before induction may explain, why induction at lower tetracycline concentration of 20 µg/ml is enough to notably enhance the expression of asRNA_154_, yet does not affect sRNA_154_ levels as strong. Conversely, at 40 µg/ml tetracycline a high asRNA_154_ induction corresponds to a significant downregulation of sRNA_154_ transcript, and consequently of the mRNAs that are stabilized by sRNA_154_. Prasse et al. (2017) observed downregulation of the transcripts *nifH, glnK_1_*, and *glnA_1_* in sRNA_154_ deletion strains, with reductions of −5.0-fold, −11.1-fold, and −3.8-fold, respectively. In our study, using an inducible knockdown approach for sRNA_154_, we observed a remarkably consistent pattern of downregulation, but with substantially higher reductions of −31.9-, −65.5-, and −19.6-fold for *nifH, glnK_1_*, and *glnA_1_*mRNA. The consistency in the pattern of downregulation across different approaches underlines the robustness of our previous findings and reinforces the role of sRNA_154_ in the N metabolism of *M. mazei* (Prasse et al. 2017). Nevertheless, the inducible knockdown reported here results in a six times stronger downregulation of *nifH, glnK_1_*, and *glnA_1_*mRNA compared to the deletion strain from Prasse et al. (2017). This effect is likely attributable to the dynamics of the inducible expression system, where sRNA_154_ is rapidly titrated following asRNA_154_ induction. In contrast, cells in the deletion strain may adapt to the continuous absence of sRNA_154_. Additionally, the cells in a chromosomal deletion strain experience high selection pressure. Due to the presence of multiple genome copies in *M. mazei*, any nonknocked-out copy rapidly restores the sRNA_154_ across all genome copies. In this study, the plasmid carrying the inducible asRNA_154_ was isolated from *M. mazei* and sequenced multiple times after induction, but no mutations were detected, indicating lower selection pressure. Besides, the inducible knockdown approach allows for the investigation of essential genes, which is not possible with chromosomal deletion. This highlights the advantage of utilizing inducible gene expression over conventional deletion mutants not only to study gene regulation, but also for induced protein production. In fact, we show the successful deployment of this induction on transcript level to hit downstream on post-transcriptional level and the use of the TetR/TetO system to generate the first inducible antisense RNA derived knockdown mutant in archaea.

### First RNAT for temperature induced translation applied in archaea

Riboswitches and RNATs are *cis*-encoded RNA regulatory elements that employ distinct mechanisms to regulate the expression of associated genes controlling key metabolic pathways and genes of temperature responsive proteins including virulence factors in bacteria (Abduljalil 2018, Sharma et al. 2022). Archaea on the other hand have never been reported as pathogens (Bang and Schmitz 2018, Mohammadzadeh et al. 2022) and in line the studied ones do not control temperature-dependent virulence gene expression. However, considering the adaptive nature of these organisms to extreme and changing environments, it is most likely that archaea might employ similar or unique mechanisms, including temperature-responsive elements, to regulate gene expression for example to regulate heat shock proteins like DnaK homologues (Macario and Macario 1994, Lemmens et al. 2018). In bacteria, the majority of RNATs are located in the 5′-UTR of mRNAs and mask RBSs by base pairing in a secondary structure at low temperatures. Melting of these structure due to increasing temperatures permits translation initiation (Narberhaus et al. 2006). RNATs are common in bacterial systems (Waldminghaus et al. 2007, Neupert et al. 2008, Kortmann et al. 2011, Roßmanith and Narberhaus 2016, Scheller et al. 2021, Pienkoß et al. 2022), but have not been employed in archaea before, to the best of our knowledge. There is a heat‐inducible promoter reported for the hyperthermophile *Sulfolobus solfataricus*, but greatly increased mRNA levels upon temperature induction hint to transcriptional regulation rather than an RNAT (Jonuscheit et al. 2003).

**Figure 2.**
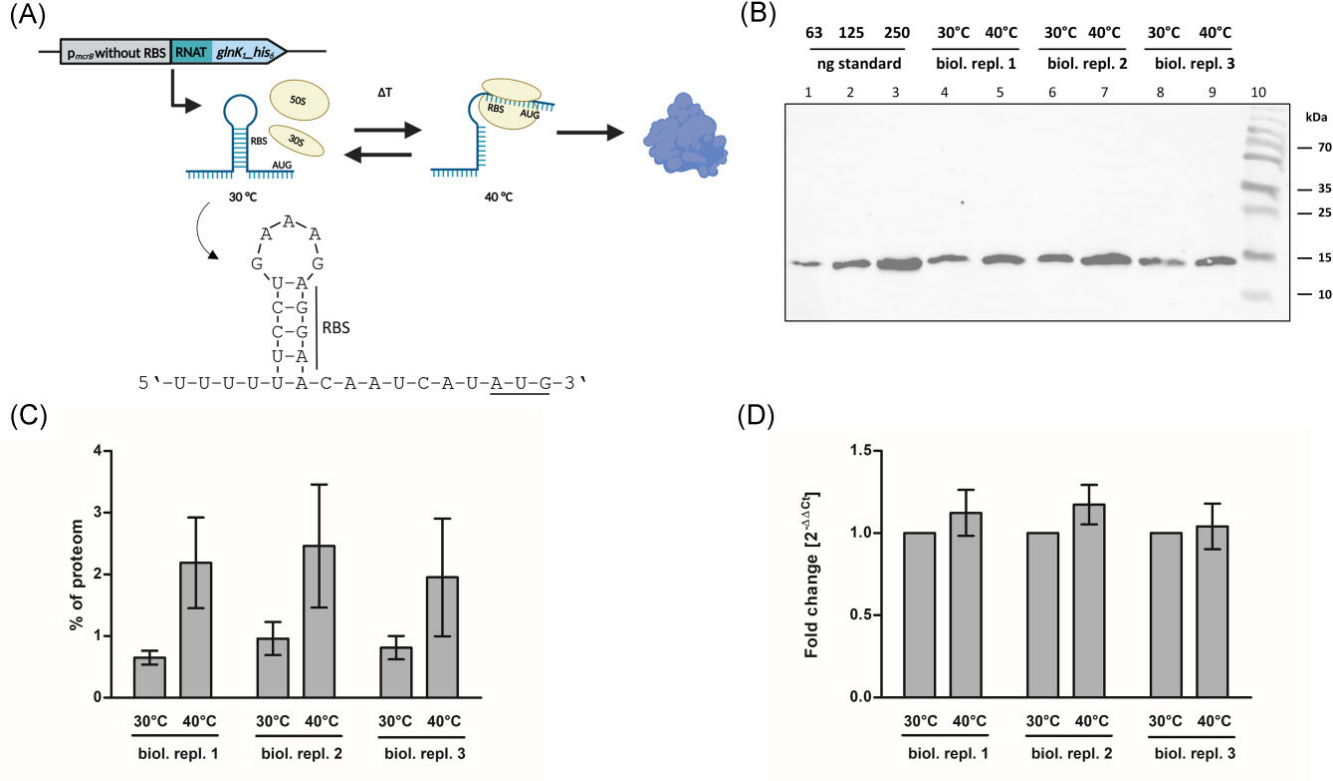
Temperature-inducible gene expression in *M. mazei*. (A) Overview of the regulation strategy: the constitutive promoter p*_mcrB_* drives overexpression of the reporter gene *glnK_1_*, with translation regulated by a 26-nt RNAT including a RBS that forms a stem-loop preventing translation at 30°C but allowing it at 40°C. (B) and (C) showGlnK_1_ protein levels before and after induction for three biological replicates, each with two technical replicates. Exponentially growing cultures at 30°C were split, and half were incubated at 40°C for 2.5 h. Western blot analysis using a primary his-tag antibody was performed on 5 µg whole cell extract. (B) GlnK_1_ protein levels in *M. mazei* at 30°C and at 40°C. Lanes 1–3: GlnK_1_ standard. Lanes 4, 6, and 8: cell extract at 30°C. Lanes 5, 7, and 9: cell extract at 40°C. Lane 10: molecular weight marker (kDa indicated on the right). Depicted is one exemplary western blot out of three technical replicates each showing three biological replicates. (C) Protein band intensities were quantified using Image Lab Software, presented as relative values from the total proteome, calculated from a standard curve. Data represent means of three technical replicates, with standard deviations as error bars. (D) Corresponding transcript levels in *M. mazei* showed no significant increase at 40°C compared to 30°C. Values were normalized using three housekeeping genes, and fold change calculated using 2^−∆∆Ct^ as described in the section ‘Materials and methods’. Data represent mean values from two technical replicates each, standard deviations are shown as error bars. Partially created with BioRender.com.

Here, we present the possibility to induce gene expression in *M. mazei* on the translational level by incorporating a reported RNAT into the 5′-UTR of a target gene for read out. Hence, the constitutive promoter p*_mcrB_* was linked to a 26-nt sequence originating from the 5′-UTR of the heat shock gene *dnaJ* from *B. melitensis* including a RBS (Waldminghaus et al. 2007). Downstream *glnK_1_* fused to a his-tag was inserted as read out resulting in plasmid pRS1966 (see Fig. 2A). To test protein expression, *M. mazei*/pRS1966 was grown at 30°C showing slower growth behaviour compared to standard conditions (see Fig. S1). Upon reaching the early exponential phase, the culture was split into two, whereof one was further incubated at 30°C, while the other one was transferred to 40°C. After 2.5 h, the cells were harvested and translation and transcription of *glnK_1_* was analysed using western blot and qRT-PCR analysis. Our results show that the reporter protein GlnK_1_ was already produced at 30°C (see Fig. 2B), however the protein amount increased when shifted to 40°C by factor 2.8 ± 0.4 (see Fig. 2B and C). The corresponding transcript levels revealed that this induction was indeed based on the translation as the transcript level remained similar (see Fig. 2D). As a control, the promoter p*_mcrB_* was used and two other potential RNATs natively located in the 5′-UTR of the *Synechocystis hsp17* transcript (Kortmann et al. 2011) and in the 5′-UTR of the *Salmonella agsA* transcript (Waldminghaus et al. 2007) were tested showing no inducible gene expression in *M. mazei* (see Fig. S2). These three RNATs were chosen from the literature as their sequences include a bacterial RBS (5′-AGGAG-3′) that can also be used for translation initiation by *M. mazei*. Notably, the archaeal translation machinery exhibits more similarities with the eukaryotic (e.g. ribosomal structure and translation factors) than with the bacterial system (Schmitt et al. 2020), which makes the use of the here reported bacterial RNAT even more valuable. In bacterial systems, the translation induction using an RNATs ranges between 2- and 12-fold (Waldminghaus et al. 2007, Kortmann et al. 2011, Scheller et al. 2021, Pienkoß et al. 2022). However, it is noteworthy that in bacteria, full induction often occurs only at 42°C, a temperature significantly stressful for *M. mazei* (see Fig. S1). Particularly when cells are cultivated at 30°C or lower temperatures, transitioning to 42°C not only triggers a severe stress response but also results in cell lysis (data not shown). Hence, there is a necessity to find additional putative RNATs capable of inducing translation with smaller temperature shifts. Despite the challenge in identifying new RNATs through bioinformatics posed by minimal sequence conservation, a recent study employs an *in silico* motif approach to computationally identify RNATs, potentially enhancing future searches, particularly within archaeal species (Sharts et al. 2024). Additionally, the entire field of synthetic RNA biology has made tremendous progress in recent years making it likely that artificially designed RNAT will become more important in the future, as scientists frequently recognize the capability of RNA to interact in a very specific and predictable way through complementary base pairing, but also exploit the ability of RNA to form highly complex structures (Suess 2024). Due to the sensitivity of RNAs to sequence changes, even minor variations can exert a profound influence on the secondary structure and stability. This might also explain, why only one out of the three tested RNATs worked in *M. mazei*. A notable illustration can be found in the investigations by Kortmann et al. (2011), where single-point mutations in the RNAT sequence were shown to significantly repress or derepress translation upon a temperature shift. These findings suggest that the herein reported RNAT might be further refined through subtle modifications, thus potentially facilitating its adaptation to use for induced gene expression in *M. mazei* and other mesophilic methanogens. Also, several single-point mutations could be introduced in the two nonactive RNATs (see Fig. S2) to investigate if small adaptations are sufficient to restore the activity that was shown in the bacterial induction systems (Waldminghaus et al. 2007, Kortmann et al. 2011). Thus, the proof of concept for the first archaeal RNAT reported here should be utilized to explore and optimize more RNATs in archaea, aiming to further enhance induced gene expression in these fascinating organisms.

### New insights in the expression of methylamine transferase genes

**Figure 3.**
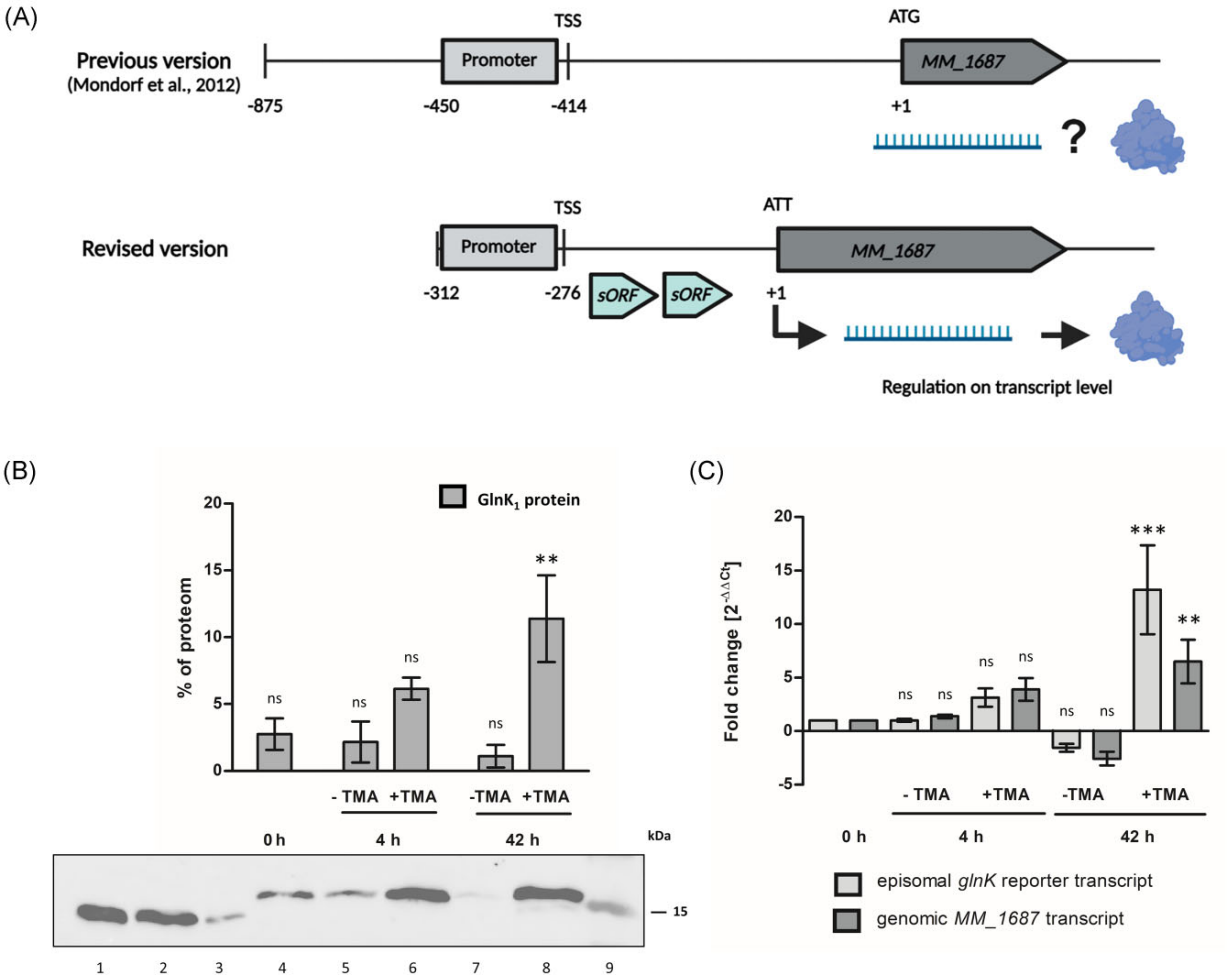
TMA-inducible gene expression in *M. mazei*. (A) Overview of the inducible *MM_1687* system: an optimized version of the long 5′-UTR of *MM_1687* mRNA, initially suggested by Mondorf et al. (2012) as an inducible system, regulates expression at the transcript level, excluding a riboswitch mechanism. New data also identified two small open reading frames (*sORF*s) within the 5′-UTR and correct a previously misannotated start codon (Tufail et al. 2024). (B and C) Cells grown with 30 mM methanol to OD_600_ of 0.2 were harvested at 0 h (control), and after 4 and 42 h following 50 mM TMA supplementation or no treatment. One part was used for western blot analysis (B), and another for RNA extraction and qRT-PCR (C). (B) GlnK_1_ expression in *M. mazei*: Lanes 1–3: GlnK_1_ standard. Lanes 4–8: cell extract before (4), after (6 and 8) or without (5 and 7) TMA induction, 10 µg whole cell extract each. Lane 9: molecular weight marker (kDa indicated on the right). Depicted is one exemplary western blot out of three biological replicates. Protein band intensity was quantified using Image Lab Software, and presented as relative values from the total proteome of the cell. Data are means from three biological replicates, standard deviations as error bars. (C) Fold change in expression of the episomal-derived *glnK_1_* reporter and the chromosomal-derived *MM_1687* transcript after TMA induction, relative to the control cultures (0 h). Values were normalized with three housekeeping genes, and fold change calculated using 2^−∆∆Ct^ as described in the section ‘Materials and methods’. Data represent mean values from three biological replicates and two technical replicates each, standard deviations are shown as error bars. Significance was calculated using a one-way ANOVA followed by a *post hoc* Tukey test with *P*-values resembled as stars with P*** < .001, P** < .01, P* < .05, and ns = not significant using GraphPad Prism version 9.4.1 for Windows, GraphPad Software, San Diego, CA, USA, www.graphpad.com (accessed on 3 May 2024). Partially created with BioRender.com.

In 2012, Mondorf et al. (2012) found that the extended 5′-UTR of *MM_1687* allowed TMA-dependent gene expression of the following operon (see Fig. 3A). Previous results demonstrated that during the degradation of methylated amines, a series of methyl transferases and corrinoid proteins (e. g. MM_1687–MM_1694) are highly active. These enzymes catalyse the stepwise demethylation of TMA to dimethylamine, and then to methylamine, and transferring the methyl groups to coenzyme M, thereby integrating them into the carbon metabolism. Their genes are organized in an operon (Krätzer et al. 2009) with the transcriptional start site being located 414 nt upstream of the annotated start codon (Jäger et al. 2009). To further explore the regulation mechanism of this 5′-UTR, we examined the upstream region of *MM_1687* (*mtbC*), postulating the potential presence of a riboswitch or other regulatory elements within its extended structured 5′-UTR (see Fig. S3). Unlike RNATs, which modulate gene expression in response to temperature fluctuations, riboswitches regulate gene expression by binding to specific ligands (e.g. metabolites), followed by changed structure, thereby regulating key metabolic pathways. This ligand-dependent control enables precise cellular adaptation to changes in nutrient availability and environmental conditions, facilitating fine-tuned metabolic regulation (Breaker 2022). For bacteria, over 55 distinct classes of natural riboswitches have been discovered, and new riboswitches are frequently found via genetic, transcriptomic, and bioinformatics searches (Kavita and Breaker 2023, Salvail and Breaker 2023). For archaea on the other hand, only few potential riboswitches were bioinformatically predicted (Weinberg et al. 2010, Gupta and Swati 2019) and even fewer were experimentally validated like the artificial tetracycline responsive riboswitch (Demolli et al. 2014), the native fluoride responsive riboswitch (Speed et al. 2018), or the synthetic theophylline-dependent translational riboswitches (Born et al. 2021).

Here, to elucidate the transcriptional and translational regulation of the operon MM_1687–MM_1694, we constructed pRS1913 containing 450 nt upstream of the start codon including a promoter (containing a BRE box and a TATA box) and the transcription start site (TSS), followed by a *glnK_1_* reporter gene with a C-terminal his-tag. *Methanosarcina mazei*/pRS1913 was grown following the protocol described by Mondorf et al. (2012), with OD_600_ ∼0.2 representing the mid-exponential phase, as cells grow to a lower OD when less methanol is added (see Fig. S5). Subsequently, control cultures (0 h) were harvested, while the remaining cultures were supplemented with 50 mM TMA or proceeded without supplementation. Cells were harvested, cell extracts were generated for western blot analysis, and RNA was purified for qRT-PCR analysis. The western blot analysis revealed that the reporter protein GlnK_1_ is already present before TMA was added (0 h), but increases significantly upon TMA addition (see Fig. 3B). This is consistent with Mondorf et al. (2012) reported findings, employing an *uidA* reporter gene, although they did not evaluate β-glucuronidase activity prior to TMA induction, instead using a promoter-less vector as a control (Mondorf et al. 2012). The here obtained qRT-PCR data demonstrate, that the transcript level of the reporter as well as the natural *MM_1687* transcript increased after TMA addition, but decreased when the cells reach stationary phase without TMA supplementation (see Fig. 3C). Krätzer et al. (2009) proposed that transcription of the *MM_1687–MM_1694* genes is downregulated in the presence of alternative substrates, such as methanol. Therefore, the here presented data are in agreement with the reported transcriptional downregulation (Krätzer et al. 2009) suggesting that the regulatory mechanism operates at the transcript level, rather than at the translational level. Besides, our data indicate that protein and transcript are present prior to TMA induction, suggesting methanol is the preferred carbon source (see Fig. S5), and upon depletion, cells seek alternatives. Indeed, increased methanol addition results in reduced induction (data not shown) indicating catabolite repression, which led us to modify the protocol to utilize only 30 mM methanol as carbon source like Mondorf et al. (2012). In the previous study, they used 875 nt upstream of the start codon to include potential regulation sites upstream of the promoter (Mondorf et al. 2012), but our investigation revealed effective induction with a construct missing the region upstream of the promoter. This shows once again the regulatory importance of the 5′-UTR and prompts speculation regarding the presence of a repressor-binding site between the promoter and the TSS and motivates further inquiry into the mechanisms governing its regulatory role. This is in agreement with recent finding of TMA-dependent transcriptional regulation of the homologous operon by a repressor protein in *M. acetivorans* (Dr Fernando Medina Ferrer and Dr Dipti Nayak, University of California, Berkeley, CA, USA, personal communication). Our recent study established Ribo-seq in *M. mazei* demonstrating two translated small open reading frames (sORFs) within the 5′-UTR of *MM_1687* (Tufail et al. 2024). These could be short upstream ORFs (uORFs), which are *cis*-acting elements located within the 5′-UTR sequence of transcripts (Silva et al. 2019). uORFs are generally considered to play a crucial role in regulating the downstream main ORF by either engaging initiating ribosomes or structurally opening the mRNA to enhance translation (Johnstone et al. 2016). Under specific environmental conditions, uORFs can become essential to activate the translation of the downstream larger ORF (Renz et al. 2020) making them key mediators of post‐transcriptional regulation (Johnstone et al. 2016). If these two reported sORFs are indeed uORFs, these might resemble additional layers of regulation of *MM_1687* on the post-transcriptional level. Furthermore, the findings of Tufail et al. (2024) suggest that the previously annotated start codon may be incorrect, indicating an earlier translation initiation site located 138 nt upstream (see Fig. 3A, Fig. S4B). In the western blot analysis, the GlnK_1_ standard proteins run according to their size at around 14 kDa, while the GlnK_1_ reporter proteins run higher in the SDS-PAGE indicating a slightly larger size (see Fig. 3B). This finding is congruent with the recent annotation of the start codon reported by Tufail et al. (2024), suggesting an increase of 46 amino acids or 4.8 kDa in the size of the reporter protein. Consequently, it signifies that the inducible promoter region utilized in this study spans only 313 nucleotides and, hence, is an applicable tool for gene expression. The here presented data are consistent with the reported transcriptional downregulation (Krätzer et al. 2009) suggesting that the regulatory mechanism operates at the transcript level. However, the Ribo-seq data from Tufail et al. (2024) hint to more complex regulation of this operon so that a regulation on translational level e.g. by sORFs cannot be excluded and should be further investigated in the future.

### Establishment of the nourseothricin resistance gene as positive selection marker

**Figure 4.**
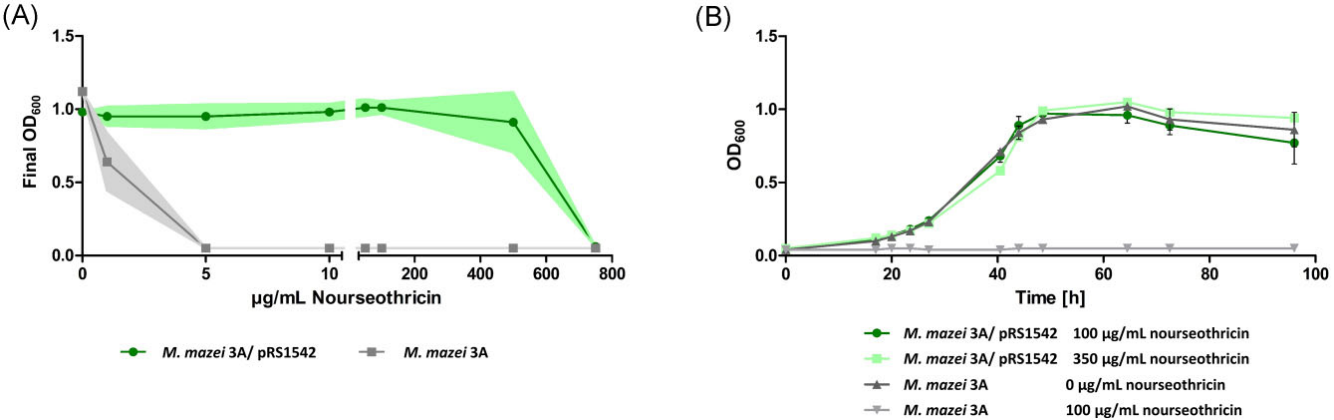
Growth behavior of *M. mazei* in the presence of nourseothricin. (A) To determine the MIC of nourseothricin, *M. mazei* wild type and a mutant strain carrying pRS1542 (expressing sat gene for nourseothricin resistance) were grown with varying nourseothricin concentrations (0–750 µg/ml). Final optical density was determined after 3–6 days. Data represent means from three biological replicates, standard deviations shown as shaded areas. (B) Growth experiments of *M. mazei* wild type and the mutant strain were conducted in 50 ml medium with nourseothricin for selection. Data represent means from three biological replicates, standard deviations shown as error bars.

Methanogenic archaea are not susceptive to a range of antibiotic compounds that effectively inhibit both bacterial and eukaryotic cells. Consequently, the identification of new selection markers suitable for genetic engineering in these organisms has proven challenging (Farley and Metcalf 2019). To test nourseothricin as a selective agent for *M. mazei*, the MIC was determined, showing that the wild-type strain was completely inhibited by 10 µg/ml and 5 µg/ml in liquid and solid media, respectively (Fig. 4A). Interestingly, these concentrations are much closer to the previously tested strains *Methanobrevibacter smithii* and *Methanomassillicoccus luminyensis* than the MIC of the closer related *M. acetivorans* and *M. barkeri* (Farley and Metcalf 2019). For *M. smithii*, spontaneous nourseothricin resistant mutants were reported and the responsible genes were identified (*msm_1096, msm_1318*, and *msm_1319*) (Farley and Metcalf 2019). These genes orthologs are absent in *M. acetivorans, M. barkeri, M. luminyensis*, and *M. mazei* possibly explaining the lack of resistant mutants in these strains. To test the *sat* gene as a positive selectable marker for *M. mazei*, plasmid pRS1542 was constructed to express the *sat* gene from *S. rochei* under the constitutive p*_mcrB_* promoter. After transformation, cells were selected with 100 µg/ml nourseothricin but show resistance against nourseothricin concentrations up to 350 µg/ml (Fig. 4B). Next, the compatibility with puromycin selection was tested by transforming a mutant carrying a *pac* gene in the chromosome with pRS1542. Transformation was performed without puromycin to reduce selection pressure, but with nourseothricin. Once cell density reached sufficient levels, they were transferred to fresh media with both puromycin and nourseothricin (data not shown). This allowed the construction of a stable double mutant carrying a chromosomal *pac* gene and an extrachromosomal *sat* gene. The findings presented demonstrate that expression of the codon optimized *S. rochei sat* gene under a constitutive promoter confers nourseothricin resistance to *M. mazei*, making it a powerful new selection marker considering the wild types susceptibility to relatively low nourseothricin concentrations.

## Conclusion

In the last decade with enhancing sustainable technologies, archaea have gained growing recognition for their biotechnological potential (Cabrera and Blamey 2018, Enzmann et al. 2018, Pfeifer et al. 2020, Lise et al. 2023) which is available now through improvements in genetics and gene expression (Kohler and Metcalf 2012, Nayak and Metcalf 2017, Fink et al. 2021). However, the absence of tightly regulated, highly responsive, and easily inducible systems in most archaeal lineages remains a major obstacle for advancing genetic research and industrial uses (Farkas et al. 2013). Researchers are addressing this challenge by developing diverse inducible gene expression systems for archaea, mostly adapted from bacterial systems (Guss et al. 2008, Demolli et al. 2014, Speed et al. 2018, Born et al. 2021). Historically, most inducible gene expression systems were protein-based on the transcription level, but with continuous improvements in techniques, equipment, and materials, laboratories are increasingly focusing on regulatory RNAs, often supplementing or replacing regulatory proteins (Morris and Mattick 2014, Bervoets and Charlier 2019). In this study, we utilized the well-characterized protein-based TetR/TetO system (Guss et al. 2008) to create an inducible antisense RNA-derived knockdown mutant. For the first time in archaea, we demonstrate an antisense knockdown, achieving significantly higher downregulation of the target transcript compared to conventional knockout mutants (Prasse et al. 2017). The combination of diverse mechanisms, potentially operating at different levels (transcription and translation) is also naturally occurring (Bervoets and Charlier 2019). Besides, we propose a complex regulation of the TMA inducible operon (MM_1687–MM_1694), which is likely regulated by a protein at the transcriptional level but may also involve additional post-transcriptional regulation, such as uORFs (Renz et al. 2020). Although our findings on this topic are tentative, we can conclude that the regulation is not riboswitch-based in the 5′-UTR. RNA-based systems like riboswitches and RNATs have gained prominence over the past decades (Roßmanith and Narberhaus 2016). While riboswitches have been occasionally adapted for archaea (Demolli et al. 2014, Speed et al. 2018, Born et al. 2021), RNATs have not been employed until now. Here, we demonstrated that a bacterial RNAT can effectively function as a translational regulation strategy in mesophilic archaea. In conclusion, noncoding regulatory RNAs are increasingly acknowledged for fine-tuning of gene expression on post-transcriptional level in bacteria (Shimoni et al. 2007, Iadevaia and Gerber 2015), and employment of more diverse regulatory strategies is crucial to fully understand the diverse group of archaea. Therefore, the toolbox reported here represents a significant step towards optimally exploiting the biotechnological potential of methanoarchaea in the future while also providing a valuable resource for advancing future genetic engineering.

## Supplementary Material

uqae019_Supplemental_Files
