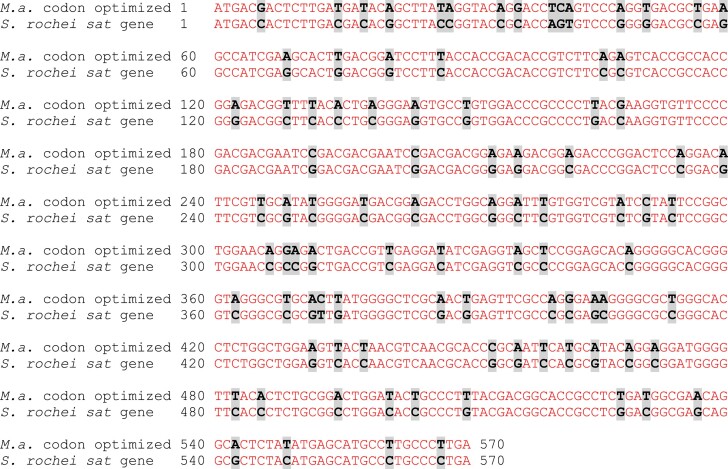# Correction to: Compiling a versatile toolbox for inducible gene expression in *Methanosarcina mazei*

**DOI:** 10.1093/femsml/uqae028

**Published:** 2025-01-28

## Abstract

Diverse capacities for hydrocarbon degradation as a function of microbial diversity in pristine oil.

This is a correction to: Johanna Hüttermann, Ruth Schmitz, Compiling a versatile toolbox for inducible gene expression in *Methanosarcina mazei, microLife*, Volume 5, 2024, uqae019, https://doi.org/10.1093/femsml/uqae019.

In the article, the authors incorrectly stated that the *sat* gene used to establish nourseothricin resistance in *Methanosarcina mazei* was non-codon optimized. In fact, the *sat* gene was synthesized with codon optimization specifically tailored for *M. acetivorans* by Dr Christian Schöne and Dr Michael Rother (TU Dresden). As a result, the sequence utilized in this study differs from the one previously published by Farley & Metcalf (2019), which was derived directly from the *Streptomyces rochei* genome.

The authors extend their gratitude to Dr Christian Schöne and Dr Michael Rother for their efforts in codon optimizing this gene and for making it available for their research.

We sincerely apologize for any inconvenience caused by our mistake.

**Figure:**