# Supplementary material for: Compiling a versatile toolbox for inducible gene expression in Methanosarcina mazei
Source: Microlife. 2024 Oct 10;5:uqae019. doi: 10.1093/femsml/uqae019 (PMC11549558; doi:10.1093/femsml/uqae019)
Supplement: uqae019_Supplemental_Files [file uqae019_supplemental_files.zip › Hüttermann&Schmitz_Supplementary data_2024_microLife_uqae019.docx]

*Supplementary data* DOI: 10.1093/femsml/uqae019

Compiling a Versatile Toolbox for Inducible Gene Expression in *Methanosarcina mazei*

Johanna Hüttermann^1^ and Ruth Schmitz^1,^*

^1^ Institute for General Microbiology, Christian-Albrechts-University, Am Botanischen Garten 1-9, 24118 Kiel, Germany

***** Correspondence: rschmitz@ifam.uni-kiel.de; Tel.: +49-8804334


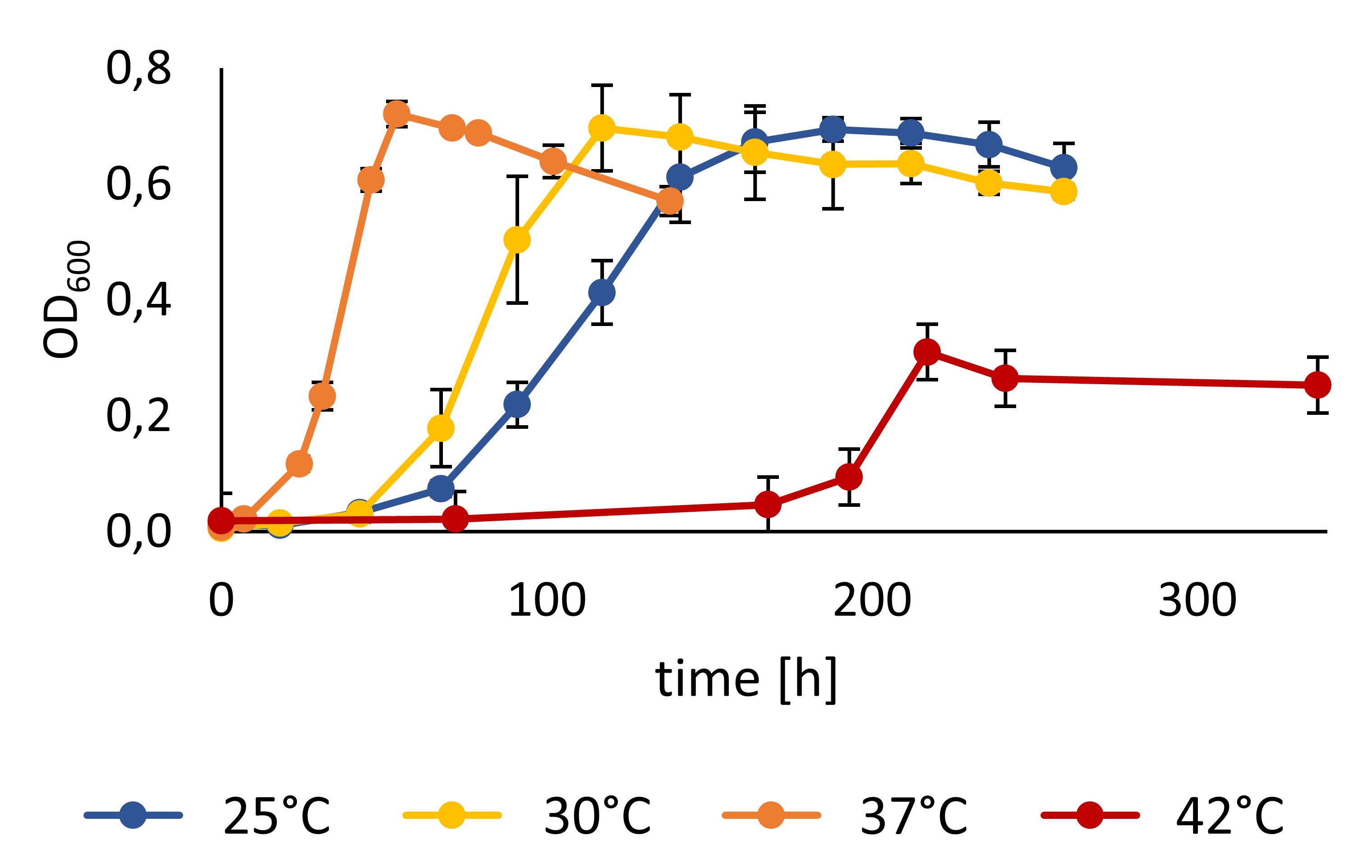


**Figure S1.** Growth behaviour of *M. mazei* wild type strain DSMZ 3647 while incubation at different temperatures. Growth experiments were conducted with 5 mL cultures in closed Hungate tubes at 25, 30, 37, and 42°C. Data represent mean values from three biological replicates, with standard deviations shown as error bars.

**Table S1.** RNA thermometer sequences. Shown is the promoter sequence with the RNA thermometers. The RNA thermometer sequence and the restriction sites are shown in bold. The sequences end with the start codon of the reporter gene *glnK_1_*.

| RNA thermometer 1  (5’-UTR of the *Synechocystis* *hsp17* transcript; Kortmann et al. 2011) | **CTCGAG**CCCTAAAAATTAAATTTTCGATTGATGCTGTTTTTTAATATAATTATAGAAAAATACAATTCACCTTAATAAAATGATTTTTAAAAAAATACATGAATTCATCTAGCGGAGAACACAAAAGATTTAAGTACCTTCTAAACGAATGAGATTTCATTGGGAAAGTGGACACTTAAAAACGACGCGGTACTTGATTTATTGAGTGCAAAAGCACTCGATTAGGTGACCAGTCCCAAAGTGATTTTAATAAATTA**ATTCAAGGGTAATCAATTCCTTCCACACATCAGGAGTTAACATTATGCATATG** |
| --- | --- |
| RNA thermometer 5  (5’-UTR of the *Salmonella agsA* transcript; Waldminghaus et al. 2007) | **CTCGAG**CCCTAAAAATTAAATTTTCGATTGATGCTGTTTTTTAATATAATTATAGAAAAATACAATTCACCTTAATAAAATGATTTTTAAAAAAATACATGAATTCATCTAGCGGAGAACACAAAAGATTTAAGTACCTTCTAAACGAATGAGATTTCATTGGGAAAGTGGACACTTAAAAACGACGCGGTACTTGATTTATTGAGTGCAAAAGCACTCGATTAGGTGACCAGTCCCAAAGTGATTTTAATAAATTA**GGACAAGCAATGCTTGCCTTGATGTTGAACTTTTGAATAGTGATTCAGGAGGTTAATGATGCATATG** |
| RNA thermometer 9  (5’-UTR of the *Brucella melitensis* gene *dnaJ* transcript; Waldminghaus et al. 2007) | **CTCGAG**CCCTAAAAATTAAATTTTCGATTGATGCTGTTTTTTAATATAATTATAGAAAAATACAATTCACCTTAATAAAATGATTTTTAAAAAAATACATGAATTCATCTAGCGGAGAACACAAAAGATTTAAGTACCTTCTAAACGAATGAGATTTCATTGGGAAAGTGGACACTTAAAAACGACGCGGTACTTGATTTATTGAGTGCAAAAGCACTCGATTAGGTGACCAGTCCCAAAGTGATTTTAATAAATTA**TTTTTTCCTGAAAGAGGAACAATCATATG** |


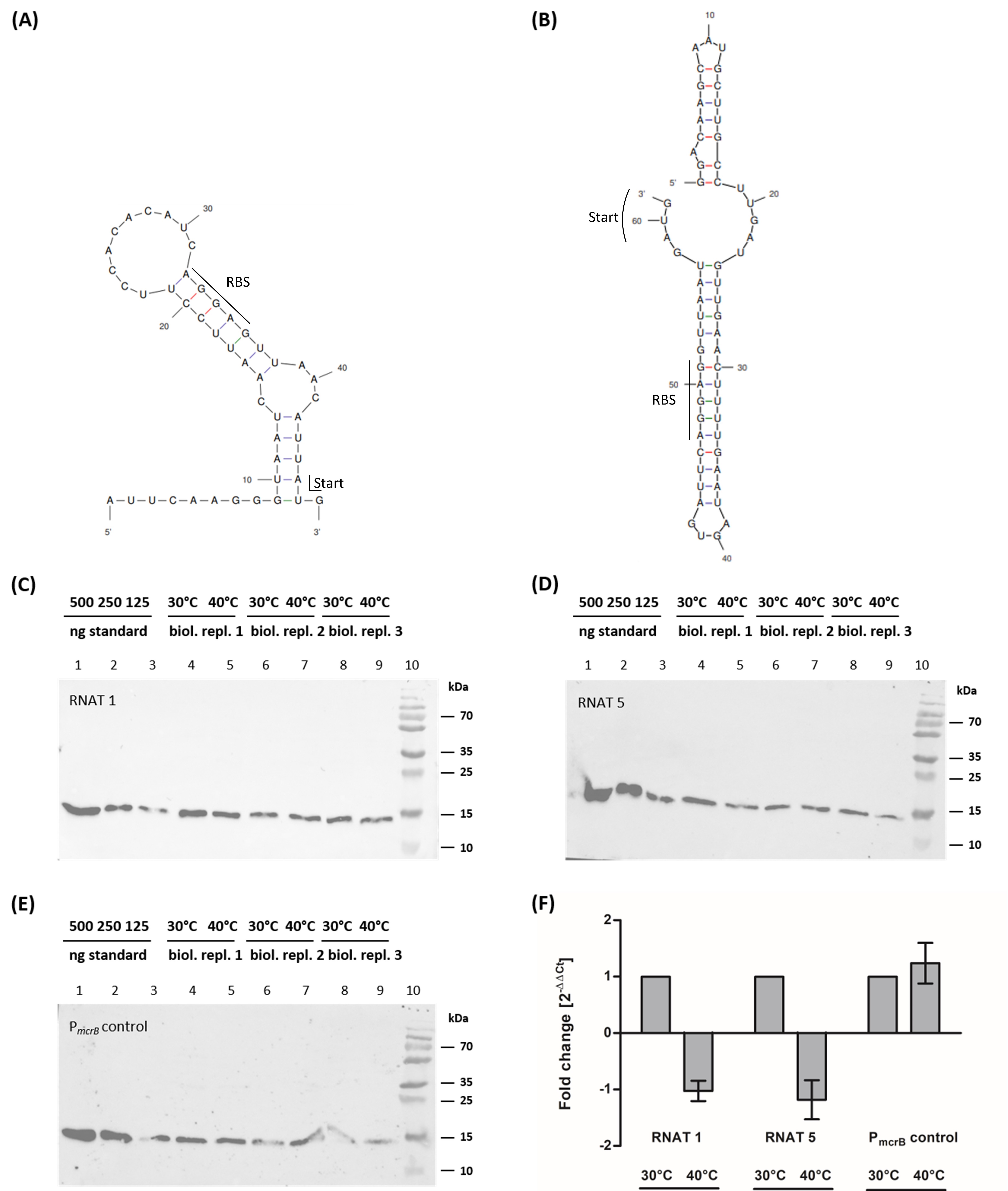


**Figure S2.** Testing reported bacterial RNA thermometers in *M. mazei* at 30°C and at 40°C. (A-B) The constitutive promoter p*_mcrB_* was used to overexpress the reporter gene *glnK_1_*. Between the promoter and the start codon a potential RNA thermometer (RNAT) including a ribosome binding site (RBS) was incorporated forming a stem-loop. The sequence was folded using Mfold web server (accessed on 03.05.24). Two different RNA thermometers natively located in the 5’-UTR of the *Synechocystis* *hsp17* transcript (Kortmann et al. 2011) and in the 5’-UTR of the *Salmonella agsA* transcript (Waldminghaus et al. 2007) were tested. (C-E) shows GlnK_1_ expression levels in *M. mazei* before and after induction for three biological replicates. Exponentially growing cultures at 30°C were split, followed by an incubation at 40°C for half of the cultures. All cultures were harvested after 2.5 h and whole cell extract was used for western analysis using a primary antibody against the his-tag. Lane 1-3: GlnK_1_ standard. Lane 4, 6, 8: cell extract of *M. mazei* grown at 30°C. Lane 5, 7, 9: cell extract of *M. mazei* grown at 40°C. Lane 10: Molecular weight marker (size in kDa indicated on the right). (F) shows the corresponding transcript levels of one exemplary replicate and demonstrates no significant fold change of reporter transcript level at 40°C in *M. mazei* compared to a control culture incubated at 30°C. The values were normalized with three housekeeping genes. Data represent mean values of one biological replicate with two technical replicates each, with standard deviations shown as error bars.


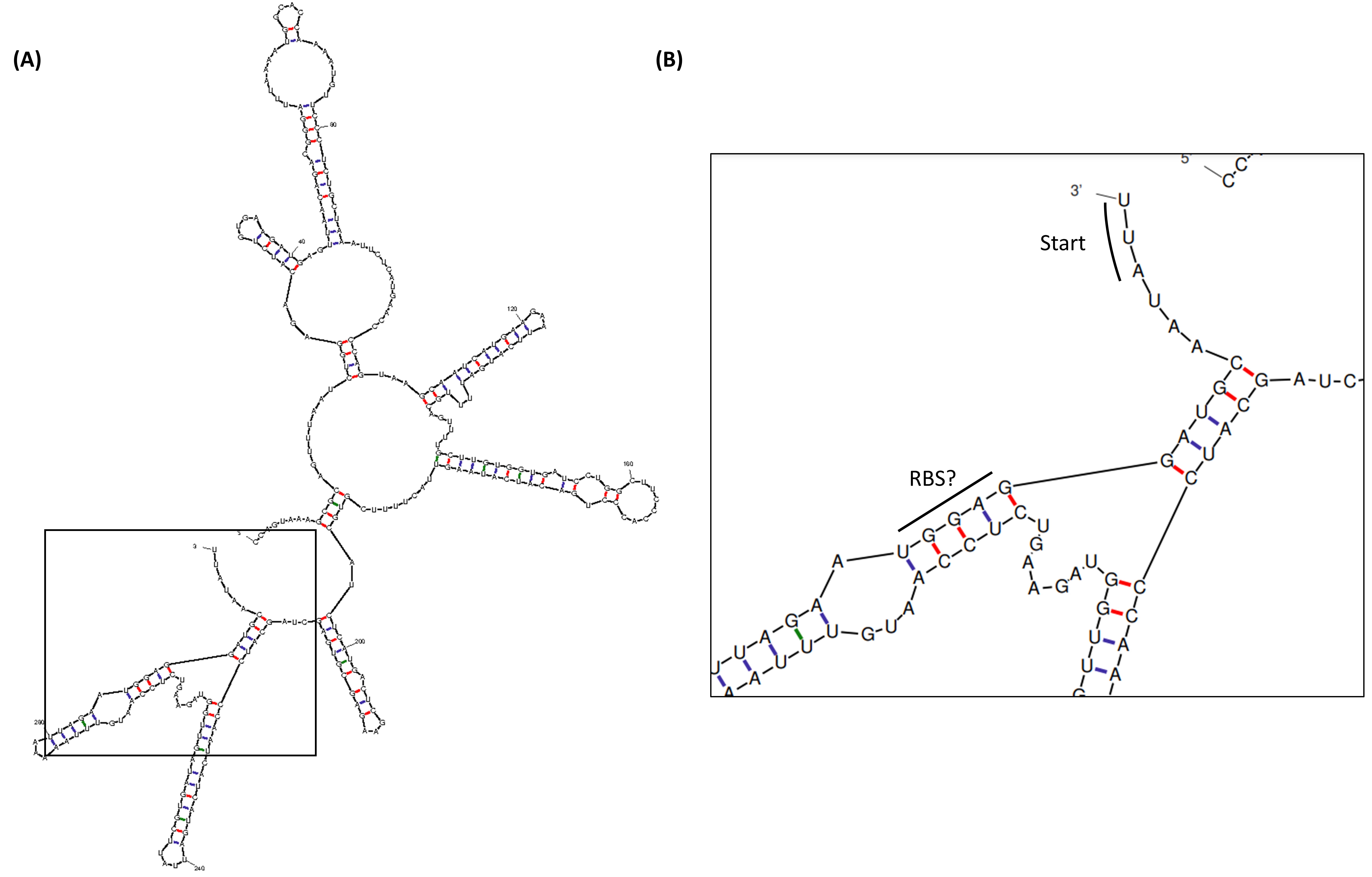


**Figure S3.** Secondary structure of the 5’-UTR of *MM_1687*. The seqeunce between the promoter and the translational start codon was folded using Mfold web server (acceced on 24.04.24).


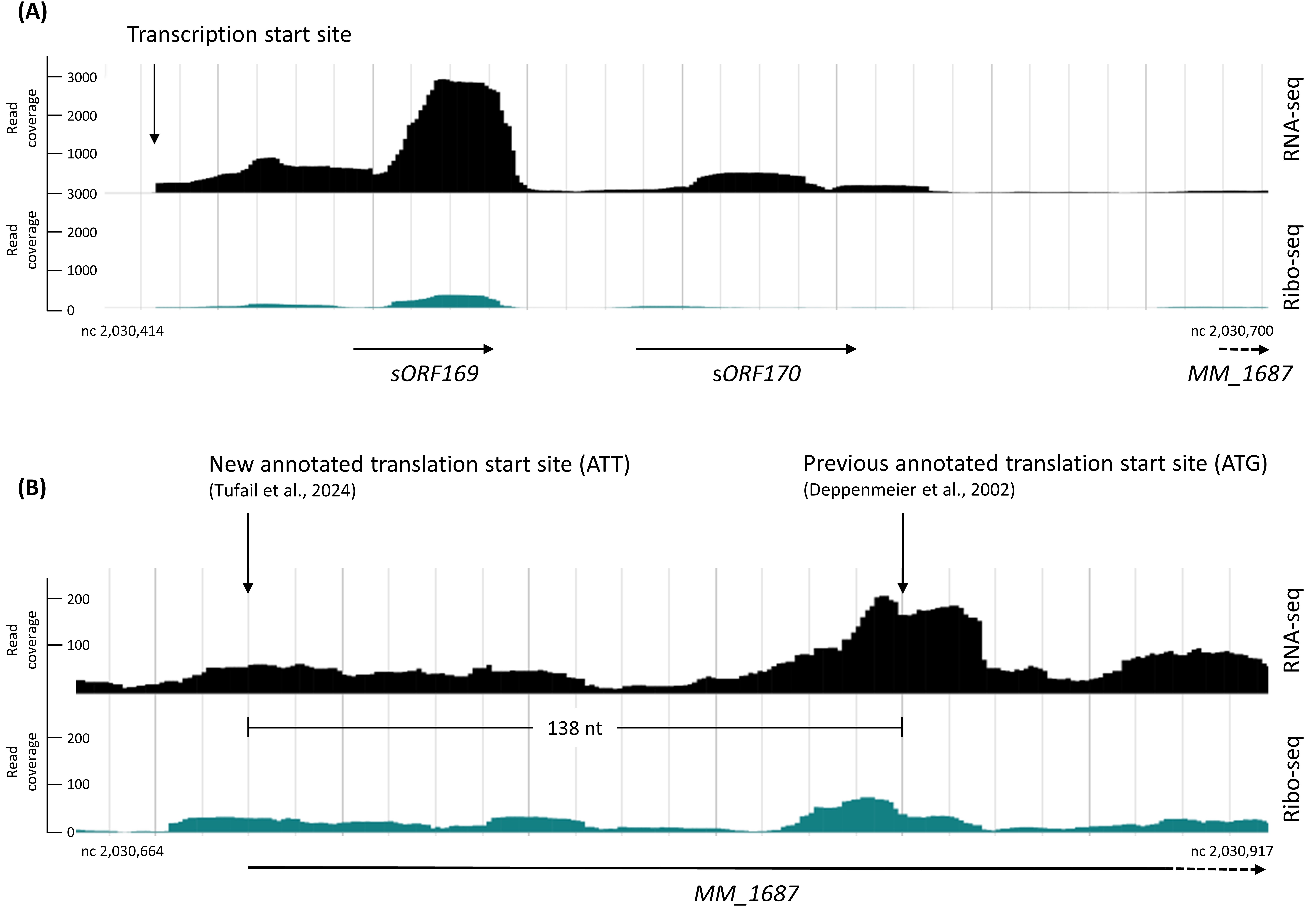


**Figure S4.** Visualization of RNA-seq and Ribo-seq data highlighting key genetic features in 5’-UTR of *MM_1687*. (A) Two newly identified small open reading frames, *sORF1* and *sORF2*, are evident in the data set. Their corresponding peaks suggest active transcription and translation (for details see Tufail et al., 2024). (B) The data also supports the revision of the start codon, indicating an alternate initiation site that is utilized. Analysis was conducted using JBrowse.


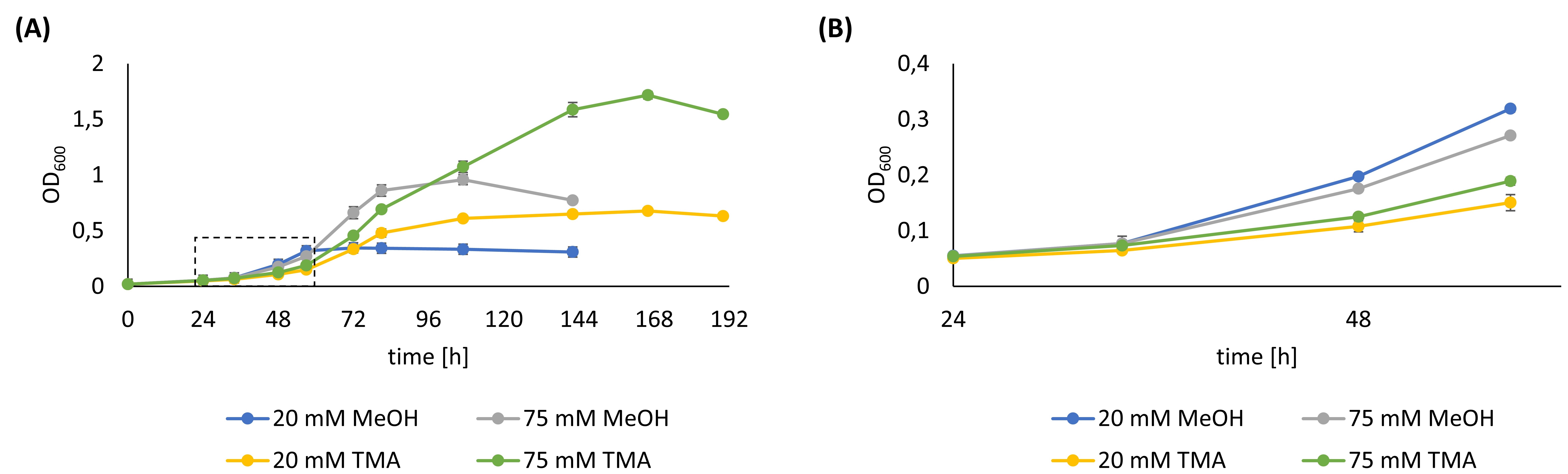


**Figure S5.** Growth behavior of *M. mazei* wild type strain DSMZ 3647 with different carbon sources. (A) presents the full growth curve of *M. mazei* cultured with either methanol (MeOH) or trimethylamine (TMA) as carbon source at 37°C. (B) offers a detailed view of the early exponential phase from time 24 to 60 h as a zoom in of (A). Growth experiments were conducted in 50 mL serum bottles. Data represent mean values from two biological replicates, with standard deviations shown as error bars.


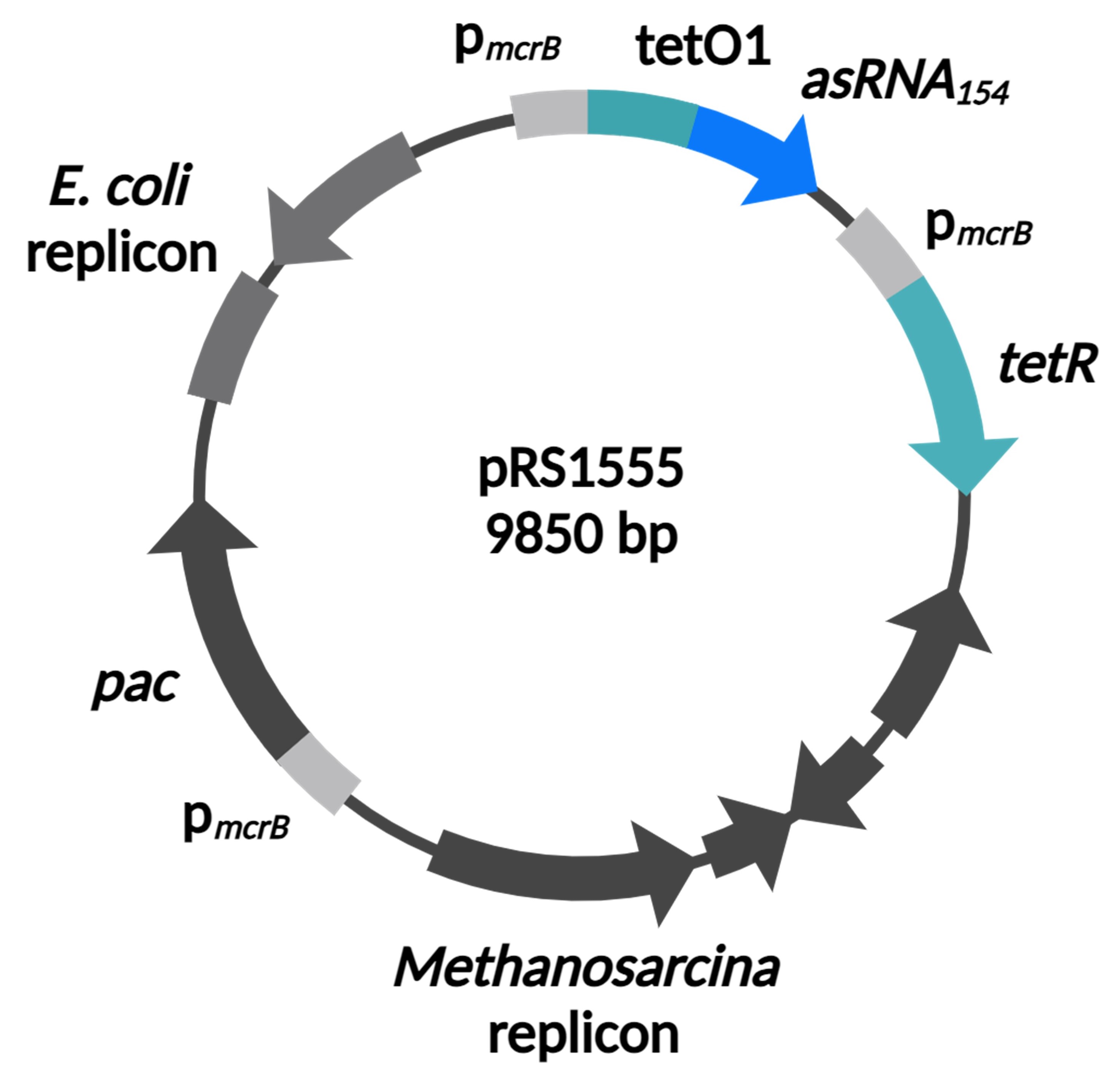


**Figure S6.** Plasmid map of pRS1555. For tetracycline inducible expression, the constitutive promoter p*_mcrB_* was modified to include a TetR binding site tetO1 (Guss et al. 2008) followed by asRNA_154_. The tetR gene was cloned under the strong constitutive promoter p*_mcrB_*. Both was cloned into the shuttle vector pWM321 (Metcalf et al. 1997) inlcuding a replicon for *Methanosarcina* species and a replicon for *E. coli*.
